# Cloning and sequencing of *hfq* (host factor required for synthesis of bacteriophage Q beta RNA) gene of *Salmonella* Typhimurium isolated from poultry

**DOI:** 10.14202/vetworld.2015.610-614

**Published:** 2015-05-14

**Authors:** Parthasarathi Behera, Muhammed Kutty, Bhaskar Sharma, Ajay Kumar, Meeta Saxena

**Affiliations:** Division of Biochemistry, Indian Veterinary Research Institute, Izatnagar, Bareilly, Uttar Pradesh, India

**Keywords:** cloning, *hfq*, RNA binding protein, sequencing, *Salmonella* Typhimurium

## Abstract

**Aim:**

The aim was to clone and sequence *hfq* gene of *Salmonella* Typhimurium strain PM-45 and compare its sequence with *hfq* gene of other serovar of Salmonella.

**Materials and Methods:**

*Salmonella* Typhimurium strain PM-45 was procured from the G. B. Pant University of Agriculture and Technology, Pantnagar, India. The genomic DNA was isolated from *Salmonella* Typhimurium. *Hfq* gene was polymerase chain reaction (PCR) amplified from the DNA using specific primers, which was subsequently cloned into pET32a vector and transformed into *Escherichia coli* BL21 pLys cells. The recombinant plasmid was isolated and subjected to restriction enzyme digestion as well as PCR. The clone was then sequenced. The sequence was analyzed and submitted in GenBank.

**Results:**

PCR produced an amplicon of 309 bp. Restriction digestion of the recombinant plasmid released the desired insert. The *hfq* sequence shows 100% homology with similar sequences from other *Salmonella* Typhimurium isolates. Both nucleotide and amino acid sequences are highly conserved. The submitted sequence is having Genbank accession no KM998764.

**Conclusion:**

*Hfq*, the hexameric RNA binding protein is one of the most important post-transcriptional regulator of bacteria. The sequence of *hfq* gene of *Salmonella* Typhimurium is highly conserved within and between *Salmonella enterica* serovars. This gene sequence is probably under heavy selection pressure to maintain the conformational integrity of its product in spite of its being not a survival gene.

## Introduction

*Salmonella enterica* serovar Typhimurium, a gram-negative, facultative anaerobe belongs to family enterobacteriaceae [1]. This organism infects a wide range of animal hosts such as humans, cattle, pigs, horses, sheep, poultry, and rodents [2]. Contaminated animal products are the principal carriers of this pathogen [3]. Salmonella species are the most important foodborne pathogen throughout the world [4]. It is a major threat for the food and livestock industry and leading cause of gastroenteritis in both humans and animals. Poultry is one of the most important reservoirs of *Salmonellae* that can be transmitted to humans through the consumption of uncooked poultry meat and eggs [4]. The emergence of multidrug resistance strains of Salmonella against the current antibiotics [5] and indiscriminate use of antibiotics in food animals [6] has been a major concern worldwide. Hence, a thorough understanding of the different aspects of bacterial genetics, metabolism as well as its physiology is required to develop effective control measures.

The RNA chaperon, *Hfq*, has a diverse role in bacterial physiology and control of gene expression within bacterial cells [7]. It was first identified as a protein required for replication of Qb RNA bacteriophage in *Escherichia coli* [8]. *Hfq* plays a pivotal role in the fitness and virulence of many pathogenic bacteria and *hfq* deletion mutants exhibit pleiotropic phenotypes such as decreased growth rate, reduced survival in stress conditions as well as reduced virulence [9]. *Hfq* has also emerged as a key factor in stabilizing small RNAs (sRNAs) and facilitating their interactions with mRNA targets [10]. About 20% of genes of Salmonella are reported to be regulated by *hfq* [9]. *Hfq* plays a significant role in biofilm formation in *Salmonella* Typhimurium [11]. This protein is also involved in the expression and secretion of virulence factors in *Salmonella* Typhimurium [12]. Despite the extensive role it plays, it is not an essential gene for the survival of *Salmonella* spp. *in-vitro* or *in-vivo*. Deletion of *hfq* though highly attenuates the Salmonella *in-vivo* [13]. The *hfq* is not a survival gene, still it is a key metabolic regulator, so the question comes whether its sequence is under selection pressure or not. In this study, we had cloned and sequenced the *hfq* gene of poultry isolate of *Salmonella* Typhimurium, analyzed and compared its sequence with other sequences and show that the sequence of *hfq* is highly conserved across species and isolates.

## Materials and Methods

### Ethical approval

The study was carried out after approval of Institutional Animal Ethics Committee of Indian Veterinary Research Institute.

### Bacterial culture and characterization

The poultry isolate of *Salmonella* Typhimurium strain PM-45 was received from Dr. Mumtesh Saxena, College of Veterinary sciences, Govind Ballabh Pant University of Agriculture and Technology, Pantnagar, India. The culture was revived by growing in LB broth at 37°C overnight. A loop-full of culture was then streaked on Hektoen enteric agar (HEA) plate to obtain typical isolated colonies of Salmonella. Further cultures were confirmed by conventional biochemical methods as well as by polymerase chain reaction (PCR) using gene-specific primer [14].

### Molecular biology techniques

The genomic DNA was isolated from the culture as per the standard protocol (purelink™ genomic DNA isolation kit, Invitrogen™, USA). The absorbance of extracted DNA was measured at 260 nm and at 280 nm in NanoDrop spectrophotometer. The ratio of OD_260_ and OD_280_ was calculated to check purity of the extracted DNA. The integrity of the DNA was also checked by running on 1% w/v agarose gel (Ultrapure™ agarose, Invitrogen, USA). The primers of *hfq* gene of *Salmonella* Typhimurium were designed using GeneTool software and their specificity was checked by Basic Local Alignment Search Tool (BLAST) (http//ncbi.nlm.nih.gov/BLAST/). The restriction enzyme (RE) sites of Bam HI and Sal I were incorporated on 5’ end of forward and reverse primers, respectively. The primer sequences are given in Table-1. The PCR was performed in a 40 µL reaction mixture consisting of 8 µL of ×5 phusion buffer, 1 µL d NTP (10 mM), 1.5 µL of each primer, 2 µL of extracted DNA, 0.5 µL phusion taq polymerase and finally volume was adjusted with nuclease-free water. Amplification was carried out in a gradient thermocycler (Applied Biosystem) with initial denaturation at 95°C for 5 min, followed by 32 cycles each of denaturation at 95°C for 45 s, annealing at 60°C 40 s, extension at 72°C for 1 min with a final extension period of 72°C at 10 min. The PCR product was checked by electrophoresis on 1.5% agarose gel containing ethidium bromide (0.5 µg/ml) under UV transilluminator.

**Table-1:** Designed primers of *hfq* of *Salmonella* Typhimurium.

Forward primer	5’GGAAGGATCCATGGCTAAGGGGCAATCT 3’
Reverse primer	5’GCGCGTCGACTTATTCAGTCTCTTCGCTGTC 3’

The PCR product was double digested with BamHI and SalI at 37°C overnight. Similarly, the pET32a (+) vector was also subjected to double digestion with BamHI and SalI to produce complementary overhangs. Then both the digested fragments of PCR amplified insert as well as the pET32a (+) vector were gel purified using PureLink quick gel extraction kit (Invitrogen) according to manufacturer’s instructions. The gel purified *hfq* gene insert was ligated into the digested and purified pET32a (+) expression vector. The ligation reaction was carried out by incubating the reaction mixture at 16°C for 1 h. A 1:5 molar ratio of the vector (pET32a) to insert (*hfq*) was used. The recombinant plasmid (pET32a-*hfq*) was transformed into chemically competent expression host *E*. *coli* BL21 pLys cells with 10 µl of ligated reaction mix as per standard protocol. The positive clones (containing the insert) were screened by colony PCR and RE digestion. The recombinant plasmid pET32a-*hfq* was purified using GeneJet Plasmid mini kit (Fermentas) according to manufacturer’s instructions. The *hfq* gene cloned in pET32a plasmid was confirmed by sequencing by using plasmid as template and T7 terminator and promoter as sequencing primers. Further confirmation of the positive clone was done by sodium dodecyl sulfate polyacrylamide gel electrophoresis after addition of isopropyl *β-D*-thiogalactopyranoside in the bacterial culture at the final concentration of 1 mM for 2 h at 37°C. Nucleotide sequences obtained by custom sequencing of cloned genes were searched for similarity using BLAST program (NCBI) and GeneTool. The sequence was submitted to NCBI Genbank.

## Results

### Salmonella culture characterization

**Figure-1:**
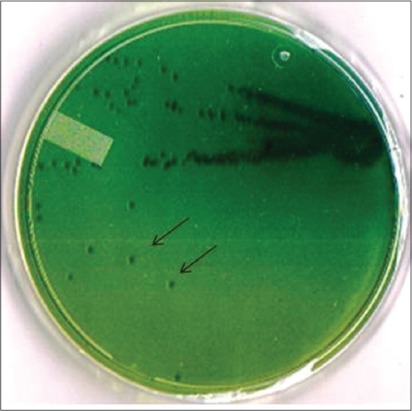
Growth of *Salmonella* Typhimurium PM-45 strain of Hektoen Enteric Agar plate. Isolated colonies are depicted by arrows.

**Figure-2:**
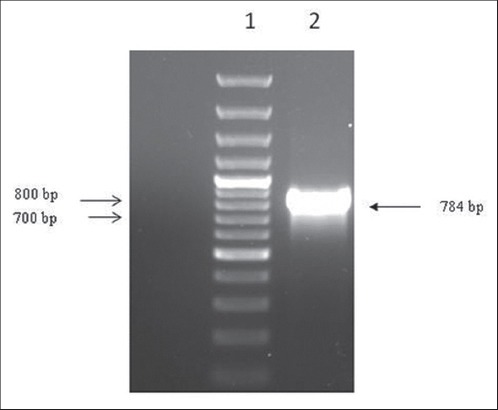
Polymerase chain reaction (PCR) detection of *Salmonella* Typhimurium Lane 1: kb DNA ladder. Lane 2: PCR amplified product marked by arrow.

*Salmonella* Typhimurium strain PM 45 used in the present study was of poultry origin. The strain showed typical morphological and biochemical characteristics. On HEA plate, the organism produced smooth, transparent, black centered colonies with greenish periphery (Figure-1). *Salmonella* Typhimurium specific PCR also generated expected size band of 784 bp (Figure-2) and thus further confirmed the isolate.

### Molecular biology characterization

**Figure-3:**
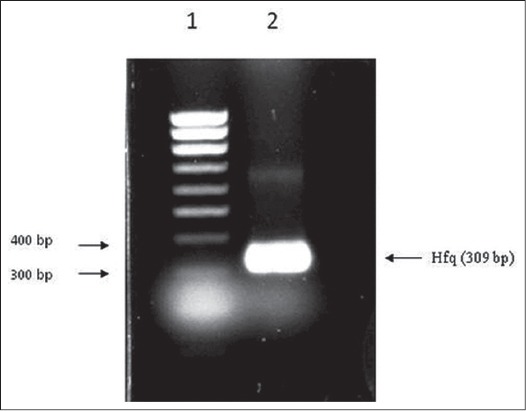
Polymerase chain reaction (PCR) amplification of *Salmonella* Typhimurium *hfq* gene using recombinant plasmid DNA as template. Lane 1: 100 bp DNA ladder, Lane 2: PCR amplified product marked by arrow.

**Figure-4:**
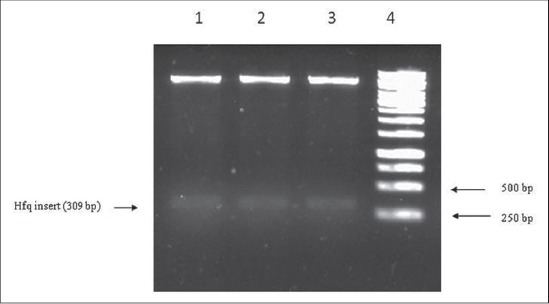
Restriction digestion of recombinant plasmid with bam H1 and Sal I showing 309 bp insert (*hfq*) release from p Et32a (+) vector. Lane 1, 2, and 3: Insert release shown by arrow, Lane 4: 1 kb DNA ladder.

The A_260_/A_280_ ratio of extracted DNA was 1.98 and it produced a sharp band above 10kb in gel electrophoresis indicating the DNA was pure and of good quality (data not shown). The PCR amplified *hfq* gene was purified from agarose gel and the concentration of the gel eluted PCR product was ~22 ng/μl. The gel purified *hfq* insert was directionally cloned into vector pET32a (+). The recombinant pET32a-*hfq*, when used as template produced the desired 309 bp band on agarose gel (Figure-3). Digesting the recombinant plasmid with Bam HI and Sal I also released the desired insert (Figure-4).

## Sequencing and Sequence Analysis

The sequencing was done through the vendor. The sequence obtained has been submitted to GenBank and has accession no. KM998764. BLAST analysis showed very high degree of similarity with other Salmonella serovars (Table-2). Our sequence and other *hfq* sequence of *Salmonella* Typhimurium (present in the Genbank) show 100% similarity. Further sequence analysis revealed that the *hfq* gene is highly conserved in other Salmonella serovars also (Table-2). Maximum sequence difference observed between *Salmonella* Typhimurium sequence and sequence from other serovars was two. These differences did not result in a change of amino acid sequence. All the changes in nucleotide were T to C or its reverse A to G.

**Table-2:**
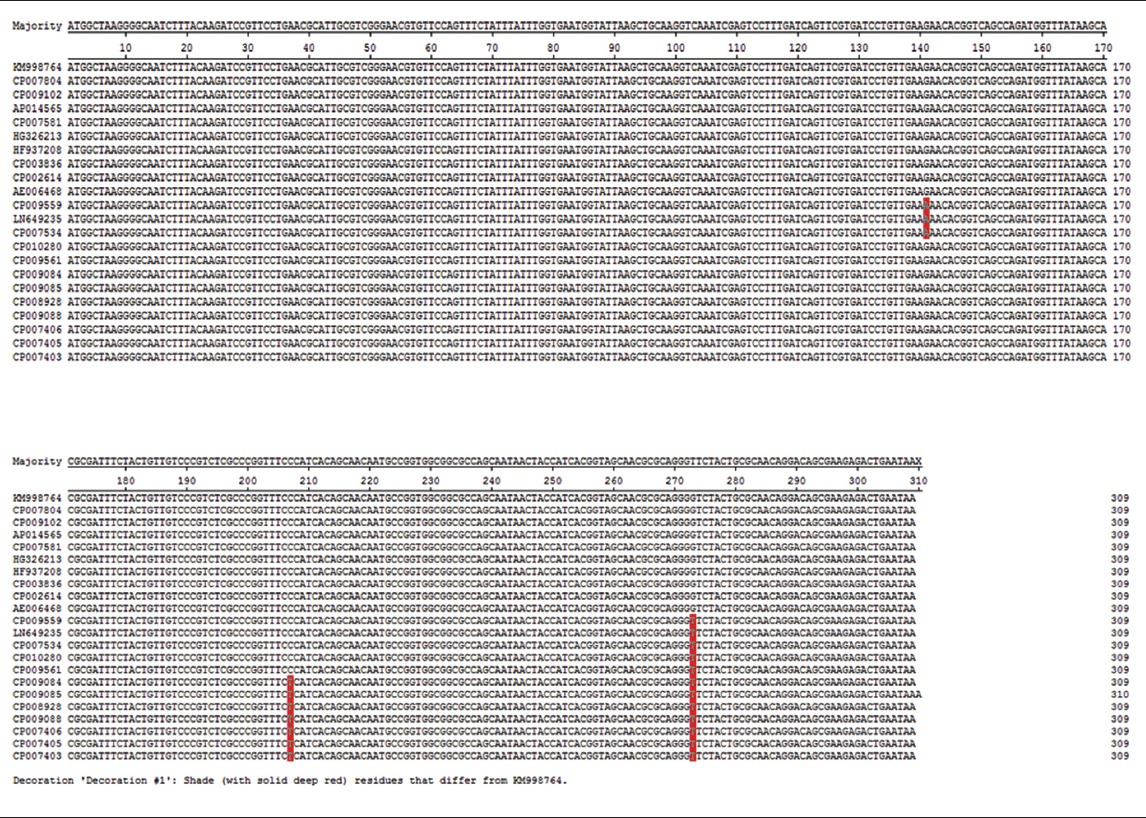
Multiple sequence alignment of *hfq* gene of *Salmonella* enterica serovar Typhimurium strain PM-45 with other Salmonella serovars.

## Discussion

The RNA chaperon *hfq* is a global post-transcriptional regulator of gene expression in large number of bacteria [7]. It was first identified in *E*. *coli* as a replication factor of coliphage and is one of the most abundant proteins in *E*. *coli* [15]. It plays a significant role in the regulation of translational activity of mRNA [16]. The role of *hfq* in sRNA based gene regulation has been well studied in *E*. *coli* [17,18]. In Salmonella, it has been reported that the *Hfq* regulates about 20% of gene expression [9]. Being an important post-transcriptional regulator, it is expected that its sequence should be highly conserved within and between species; this has been confirmed by many studies [7]. In this study, we found that the *hfq* sequence within *Salmonella* Typhimurium isolates is highly conserved. No sequence change was observed within Typhimurium isolates. These isolates have been obtained from different parts of the world and at different times. This is surprising since normal error of DNA polymerase (10^−4^) would introduce at least some error without affecting changes in amino acid. The small size of *hfq* gene (309 bp) could be the reason we didn’t find any change in the nucleic acid sequence of the *hfq* gene. The other reason could be high selection pressure due to structural constraint to maintain the functional activity of the protein. When the sequence of *hfq* of *Salmonella* Typhimurium was compared with the other serovars of Salmonella we found a single base change in two isolates (Acc. no. CP010280, CP009561) and two base changes between *Salmonella* Typhimurium and Enteritides isolates. These nucleotide changes did not change amino acid sequence indicating once again high selection pressure to maintain structural integrity of the *hfq* protein. The sequence analysis data indicating high structural integrity is also supported by the functional requirement of *hfq* protein. It thus appear, that being a small multimeric and an RNA binding protein, the *hfq* protein does not have the structural flexibility to tolerate even small changes in its structure without affecting its function. These functional and structural constraints probably are the attributes the reasons for high selection pressure to maintain its sequence.

## Conclusion

*Salmonella* Typhimurium, the prime etiological agent of nontyphoidal salmonellosis is the most important pathogenic bacteria of poultry. It causes major economic loss to the poultry industry. The RNA binding protein *HFQ* is one of the major post-transcriptional regulators of Salmonella gene expression. Our sequence analysis data revealed that the *hfq* gene is under high selection pressure to maintain the structural and functional integrity of the protein. The probable reasons for this high selection pressure could be the RNA binding activity, small size, and multimeric nature of the protein.

## Authors’ Contributions

PB and BS designed the experiment. PB, MK conducted the experimental work. PB, BS, AK, and MS were involved in scientific discussion and analysis of the data. PB and BS drafted and revised the manuscript. All authors read and approved the final manuscript.
